# Supplementary material for: Identification of photoperiod-induced specific miRNAs in the adrenal glands of Sunite sheep (Ovis aries)
Source: Front Vet Sci. 2022 Jul 22;9:888207. doi: 10.3389/fvets.2022.888207 (PMC9354845; doi:10.3389/fvets.2022.888207)
Supplement: Supplementary file 1 [file Data_Sheet_1.ZIP › Supplementary file/miRDeep2 file.zip!/miRDeep2 file/Know_Structure/SP42A3/oar-mir-495.pdf]

miRBase precursor : oar-mir-495
Total read count : 7961
oar-miR-495-5p read count : 52
oar-miR-495-3p read count : 7904
remaining reads : 5

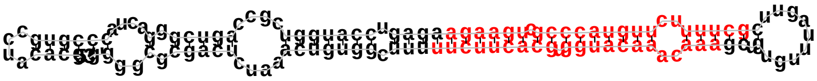

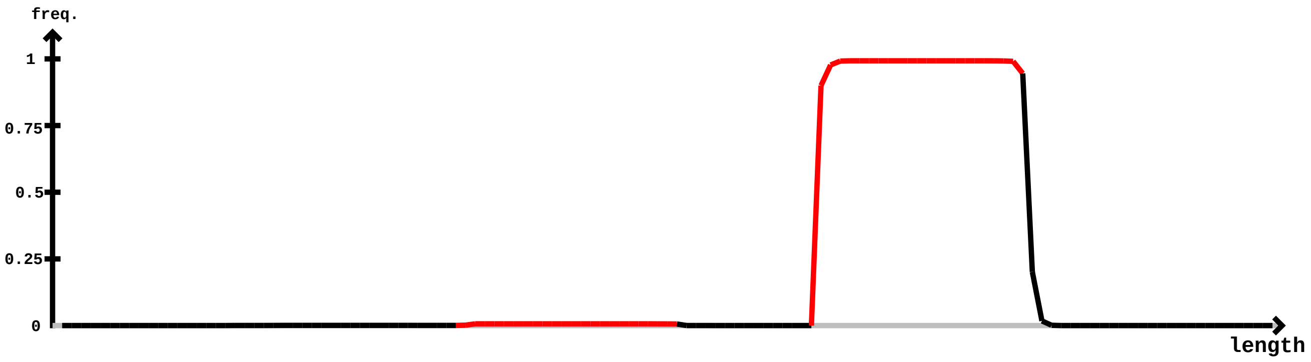


```
                                                                               oar-miR-495-3p
                                         oar-miR-495-5p
5'- gcacauccgugcccaucagggcugaccgcugguaccugagaagaagucgcccauguucuuuucgcuugauuugugucgaaacaaacauggugcacuucuuuucggugucaaaucucagcgcgggg  -3'   exp
    ((((....))))((....(.(((((....(((((((.((((((((((.(((((((((..((((((.........).)))))..))))))).))))))))))))).)))))))....))))).)..))  reads  mm
    .................agggcugaccgcugguacc..........................................................................................  1      0
    .................agggcugaccgcugguaccugaga.....................................................................................  1      0
    .....................cugaccgcugguaccugagaa....................................................................................  1      0
    ......................ugaccgcugguaccugagaa....................................................................................  2      0
    .........................................agaagucgcccauguucuuuu................................................................  1      0
    .........................................agaagucgcccauguucuuuuc...............................................................  1      0
    .........................................agaagucgcccauguucuuuucg..............................................................  7      0
    ..........................................gaagucgcccauguucuuu.................................................................  1      0
    ..........................................gaagucgcccauguucuuuu................................................................  1      0
    ..........................................gaagucgcccauguucuuuuc...............................................................  1      0
    ..........................................gaagucgcccauguucuuuucg..............................................................  39     0
    ..........................................gaagucgcccauguucuuuucgcuu...........................................................  1      0
    ..............................................................................aaacaaacauggugcacu..............................  1      0
    ..............................................................................aaacaaacauggugcacuu.............................  3      0
    ..............................................................................aaacaaacauggugcacuuc............................  11     0
    ..............................................................................aaacaaacauggugcacuucu...........................  336    0
    ..............................................................................aaacaaacauggugcacuucuu..........................  5699   0
    ..............................................................................aaacaaacauggugcacuucuuu.........................  1076   0
    ..............................................................................aaacaaacauggugcacuucuuuu........................  37     0
    ...............................................................................aacaaacauggugcacuuc............................  1      0
    ...............................................................................aacaaacauggugcacuucu...........................  22     0
    ...............................................................................aacaaacauggugcacuucuu..........................  216    0
    ...............................................................................aacaaacauggugcacuucuuu.........................  352    0
    ...............................................................................aacaaacauggugcacuucuuuu........................  24     0
    ...............................................................................aacaaacauggugcacuucuuuuu.......................  1      0
    ................................................................................acaaacauggugcacuucu...........................  3      0
    ................................................................................acaaacauggugcacuucuu..........................  9      0
    ................................................................................acaaacauggugcacuucuuu.........................  36     0
    ................................................................................acaaacauggugcacuucuuuu........................  66     0
    ................................................................................acaaacauggugcacuucuuuuu.......................  3      0
    .................................................................................caaacauggugcacuucuuuu........................  3      0
    .................................................................................caaacauggugcacuucuuuuu.......................  5      0
```
